# First steps in modelling turbulence and its origins: a commentary on Reynolds (1895) ‘On the dynamical theory of incompressible viscous fluids and the determination of the criterion’

**DOI:** 10.1098/rsta.2014.0231

**Published:** 2015-04-13

**Authors:** Brian E. Launder

**Affiliations:** School of Mechanical, Aerospace and Civil Engineering, The University of Manchester, Manchester, UK

**Keywords:** turbulent flow, laminar-turbulent transition, Reynolds averaging, computational fluid dynamics

## Abstract

Reynolds' paper sought to explain the change in character of flow through a pipe from laminar to turbulent that his earlier experiments had shown to occur when the dimensionless group that today bears his name exceeded approximately 2000. This he did by decomposing the velocity into mean and fluctuating components and noting how the average kinetic energy generation and dissipation rates changed with Reynolds number. The paper was only grudgingly accepted by two very distinguished referees and initially raised little external interest. As years went by, however, the averaged form of the equations of motion, known as the Reynolds equations (which were an intermediate stage in Reynolds' analysis) became the acknowledged starting point for computing turbulent flows. Moreover, some 50 years after his paper, a refinement of his strategy for predicting transition was also successfully taken up. For some engineering problems, the continual rapid growth of computing resources has meant that more detailed approaches for computing turbulent flow phenomena can nowadays be employed. However, this growth of computing power likewise makes possible a Reynolds-averaging strategy for complex flow systems in industry or the environment which formerly had to adopt less comprehensive analyses. Thus, Reynolds' approach may well remain in use throughout the present century. This commentary was written to celebrate the 350th anniversary of the journal *Philosophical Transactions of the Royal Society*.

## 1. Background

The origins of Osborne Reynolds' paper ‘On the dynamical theory of incompressible viscous fluids and the determination of the criterion’ [1] may be traced to the publication of his earlier and, in some respects, equally influential paper published in the *Philosophical Transactions of the Royal Society* a dozen years before, ‘An experimental investigation of the circumstances which determine whether the motion of water shall be direct or sinuous and the law of resistance in parallel channels’ [2]. In that contribution, which examined the flow of water through pipes, both by way of flow visualization and by comparing pressure drops along pipes of different diameter, Reynolds was able to show that ‘steady direct [fluid] motion in round tubes is stable or unstable according as [to whether] *ρUD*/*μ*> 1900 or <2000, the number being thus a criterion of the possible maintenance of sinuous or eddying motion’ [1]. That dimensionless group is, of course, now known as the *Reynolds number*, following the proposal of Prandtl [3]. This 1883 manuscript had been submitted to the *Philosophical Transactions* and been favourably reviewed by the senior figures of Sir George Stokes and Lord Rayleigh. The latter, however, as the culmination of his brief review, ended with: ‘In several places the author refers to theoretical investigation whose nature is not sufficiently indicated.’ This rather lofty observation serves as a starting point for an examination of the 1895 paper which directly took up Rayleigh's implied challenge. First, however, it will be helpful to look further back to glimpse some of the developments in Reynolds' life from which the mature engineering scientist emerged.

Osborne Reynolds had been born in Belfast in 1842 during a brief period when his father was principal of the First Belfast Collegiate School in Donegall Place. Soon after the family returned to England, however, his mother had died following the birth of Osborne's younger brother, Edward. His father never re-married and, indeed, played a very active role in his sons' development, in particular, sending Osborne for training in practical engineering practices and skills at Edward Hayes' small engineering and boat-building company in Stony Stratford. From there Reynolds proceeded to read mathematics at Queens' College, Cambridge, graduating in 1867 with a high first class honours degree. He then initially took up an appointment with engineering consultants Lawson & Mansergh in London. Within a year, however, Owens College, Manchester advertised the creation of one of the first chairs in engineering in England and Reynolds felt impelled to apply. Details of the controversies surrounding the creation of this chair and the choice of the appointee may be found in Launder & Jackson [4]. Suffice it to say here that, despite his youth and relative lack of experience, Reynolds was duly appointed; and there he remained until his retirement in 1904.

Owens College, founded in 1851, was initially located in premises on Quay Street in central Manchester in a building formerly the home of Richard Cobden, MP. By the time of Reynolds' arrival in 1868, it had already become overcrowded and there was no scope for providing the new professor with laboratory space. At least partly for that reason, his initial research was directed at explaining external physical phenomena: among others, the tails of comets, the solar corona and the aurora, and the calming of seas (by raindrops or an oil film on the surface). Following the College's move in 1873 to new buildings at the present site of the University of Manchester, Reynolds' research underwent a shift to more engineering themes, publishing, *inter alia*, a detailed patent specification on the multi-staging of turbines and two papers on heat-transfer phenomena in one of which he proposed the connection between convective heat transfer and fluid drag at the surface known today as *Reynolds Analogy*. In this paper [5], his writings already showed clear insight into the physical mechanism of turbulent mixing:

The heat carried off by air, or any fluid, from a surface…is proportional to the internal diffusion of the fluid at and near the surface…which depend[s] on two things: the natural internal diffusion of the fluid [and] eddies caused by visible motion which mixes the fluid up and continually brings fresh particles into contact with the surface. The first of these, molecular diffusion,…may be said to depend only on the nature of the fluid. The second, the effect of eddies, arises entirely from the motion of the fluid, and is proportional both to the density of the fluid and the velocity with which it flows past the surface.

By the second half of the 1870s, the very broad discipline of Fluid Mechanics had certainly emerged as his principal field of interest in which he tackled diverse fundamental problems such as the progression of dispersive waves in deep water and the motion of vortices. A summary of all these publications is provided in Jackson [6].

In 1877, after less than a decade in the post, he was elected a Fellow of the Royal Society, an achievement made all the more remarkable by the difficulties with which he had had to cope in his personal life. In 1868, shortly after his successful Chair interview, he had married Charlotte Chadwick. His joy at this event was short-lived, however, for Charlotte was to die the following year of a peritonal infection 12 days after delivering their only child, a son also named Osborne. Thus, from near the start of his appointment, Reynolds had had to deal with the added responsibilities of bringing up his son alone and without the love and support of a partner in managing family matters. Moreover, his son did not enjoy robust health and, despite his father's caring attention, died at St Leonards-on-Sea in September, 1879. A little over 2 years later Reynolds married Annie Charlotte Wilkinson, 17 years his junior. Their marriage appears to have been a happy one, producing over the years that followed three sons and a daughter. Perhaps, equally important for Reynolds, he had someone to free him of the responsibilities of household management.

It was in the halcyon period immediately following his second marriage that Reynolds' extensive exploration of flow through pipes had been undertaken [2]. The results enabled him to conclude that whether or not the flow through the pipe was *direct* or *sinuous* (or, as nowadays we should say, *laminar* or *turbulent*) depended, as indicated above, on the magnitude of the Reynolds number of the flow. The paper won widespread approval. Indeed, the two referees of the paper made laudatory references to it in subsequent speeches. Sir George Stokes served as President of the Royal Society from 1885 to 1890 and in this capacity presented Reynolds with the Society's Royal Medal in 1888 ‘for his investigations in mathematical and experimental physics and on the application of scientific theory to engineering’. More than half of Stokes' commendatory address was given over to a summary of the 1883 paper. Before that Lord Rayleigh, in his 1884 presidential address to the British Association in Montreal, likewise commended Reynolds' discoveries and added: ‘In spite of the difficulties…we may hope to attain before long to a better understanding of a subject which is certainly second to none in scientific as well as practical interest.’

## 2. The 1895 paper: the referees' perceptions and the author's response

**Figure 1.**
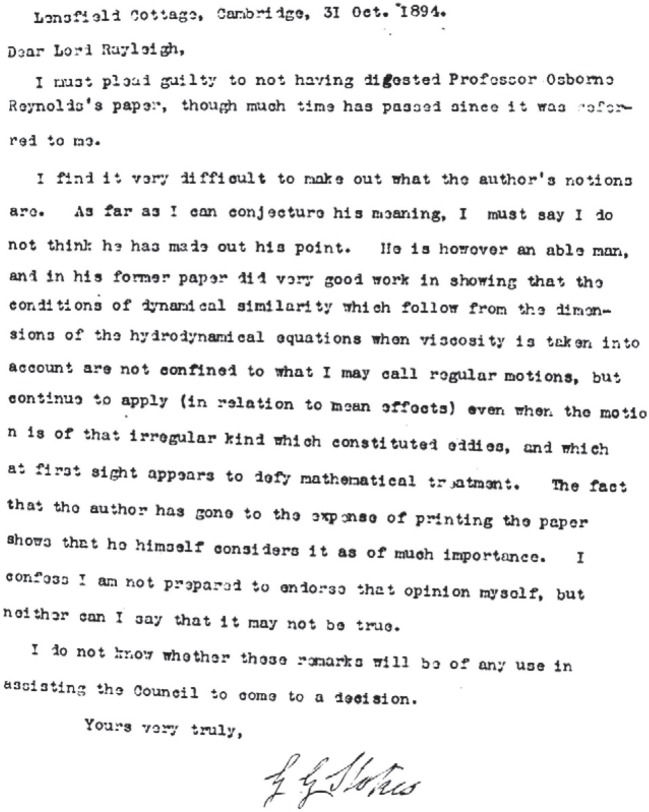
Sir George Stokes' initial response to the request to review Osborne Reynolds' paper. Copyright The Royal Society.

A full decade was to elapse before Reynolds felt ready to respond to Lord Rayleigh's observation that, while his experimental results were valuable, there was a need for a theoretical explanation. Finally, on 24 May 1894, Reynolds read the outcome of his protracted considerations to a meeting at the Royal Society. Shortly before that date he had had copies of his paper printed and had forwarded a number of them to the editor of *Philosophical Transactions*to be considered for publication. In fact, Lord Rayleigh had in the meantime become the editor of the journal and, naturally, he sent the paper for review to Sir George Stokes, who, having completed his term of office as President of the Royal Society, had returned to normal academic life. This time, however, unlike his enthusiastic review of the 1883 paper, Stokes, at the age of 75, was both equivocal and perplexed. Figure 1 shows a photograph of his typed letter of 31 October, a classic example of sitting on the fence. While his concluding sentence seems to imply that his responsibilities had been discharged, however inadequately, Rayleigh had other ideas. But on 5 December, Stokes again responded that he was ‘not yet able to go beyond the rough indication contained in a letter sent some time ago’.

In the meantime, the editor had sent the paper to a second referee, Horace Lamb, who held the Beyer Chair in Pure & Applied Mathematics at Owens College. Lamb had been elected a Fellow of the Royal Society in 1883, while a professor in Adelaide where he had published the first edition of what was probably the first English-language textbook on fluid mechanics (and which in 1895 would re-appear, greatly expanded, as Lamb's *Hydrodynamics*—a word pair that even in the twenty-first century strikes a resonance of familiarity with many fluid mechanists). Today, it would seem decidedly strange for an editor to seek a review from a colleague of the author (at that time Owens College had only five professors across the physical sciences). Lamb did not demur, however; his longhand assessment of 21 November began with the brisk summarizing statement:

I think the paper should be published in the Transactions as containing the views of its author on a subject which he has to a great extent created, although much of it is obscure, and there are some fundamental points which are not clearly established.

There followed three pages of criticism mainly concerned with preliminary matters such as the precise meanings to be attached to Reynolds' terms ‘mean-mean motion’ and ‘relative-mean motion’.

The next developments entail a degree of conjecture, for some of the documents held by the Royal Society are undated. A probable sequence is that, having received Stokes' apology of 5th December, Rayleigh (having by then received Lamb's review) persuades the two referees to communicate with one another in the hope, no doubt, of stimulating a review from Stokes. An outcome of this is that Stokes *does* provide an undated two-page review covering much the same areas of the paper as Lamb. Rayleigh, at that stage must have decided that a *joint* report would be preferable; thus, finally, on 30 January 1895, Stokes writes:

Dear Lord Rayleigh,

I enclose what Lamb meant for a draft of remarks to be submitted to the author. I think we are both disposed to say let the paper be printed, but first let some remarks be submitted to the author. There was very good work in the former [1883] paper and there may be something of importance in this, but the paper is very obscure. In its present state it would hardly be understood.

    Yours very truly,

    G. G. Stokes (Signed)

**Figure 2.**
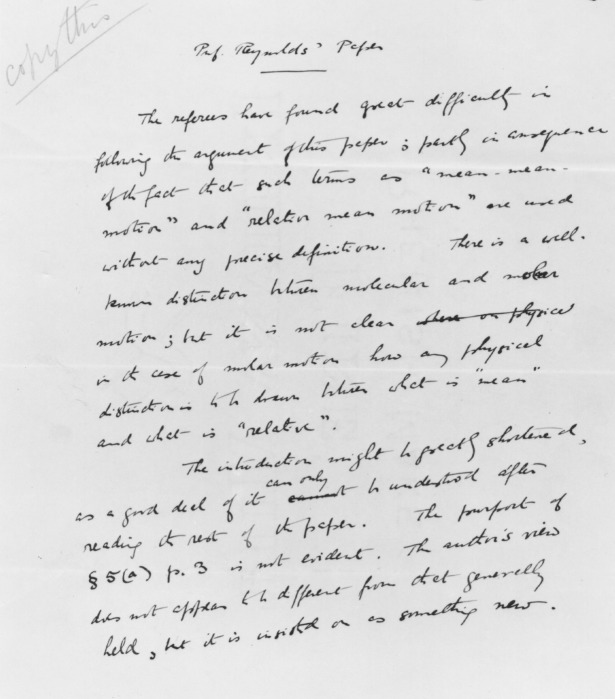
Part of the joint response of Sir George Stokes and Professor Lamb to Professor Reynolds Paper. Copyright The Royal Society.

Lamb's joint review, the first page of which appears in figure 2, largely re-states his own original assessment. The faint words ‘copy this’, visible in the top left-hand corner of the page, is Rayleigh's instruction to a clerk that the review should be transcribed prior to transmission to the author. The second paragraph begins with the sentence ‘The introduction might be greatly shortened, as a good deal of it can only be understood after reading the rest of the paper’, a proposal to which Reynolds responds in an unforeseen way, as will be seen below. The final paragraph of the review identified the key results as Eqns (15–19) and commented that: ‘If these are clearly established then a great point would be secured, but its reasoning is somewhat obscure, and needs much amplification.’

**Figure 3.**
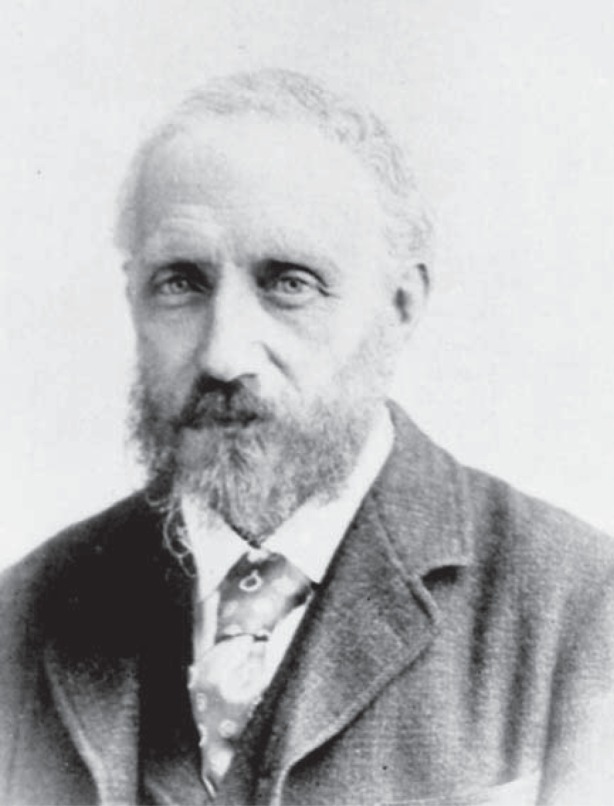
Osborne Reynolds at approximately the time of the 1895 paper. Copyright The University of Manchester.

The report was duly sent to Reynolds whose contemporary photograph appears in figure 3. After duly considering the criticisms, on 19 February, he sent a response to the editor together with a supplement to a part of the paper. Reynolds' complete handwritten letter appears, together with the reviews, under the electronic supplementary material. If his handwriting proves impossible to read in places, complete transcriptions appear in [4] and [7]. A few salient fragments from these are quoted here, beginning with:

Dear Lord Rayleigh,

From the copy of the remarks on my paper on the criterion, which you sent me, it is clear that the referees have found great difficulty in understanding the drift of the main argument.…I am very glad to know of these difficulties and the opportunity it afforded me of improving the paper in that particular.

He then continues:

That I should have scamped the preliminary explanation of this part of the argument and diffused it over the whole paper I can only explain as a consequence of its definite character having blinded me to the difficulties which would thereby result in distinguishing what was new from what was already accepted.

before announcing:

I now enclose you in M.S.S. a full preliminary description of this part of the argument which by permission I shall be glad to substitute for the first two lines of §5 p.3. It contains what I hope will be found, a clear definition of the terms mean-mean motion and relative-mean motion as well as of mean-motion …

Finally, Reynolds acknowledged ‘various misprints in the paper which must have increased the inherent difficulties of the subject’. Presumably these errors had been marked on a further printed copy of the original manuscript and returned with his letter. No further exchanges between author and editor appear to exist though the additional passage to which Reynolds refers above is plainly evident in the published paper. At the beginning of §5 is a block of material extending over more than three pages placed within square parentheses and ending with the date, Feb. 18, 1895, that is, the day prior to Reynolds' response to Lord Rayleigh.

## 3. The 1895 paper: its major contributions

Given the considerable status of the two referees and their unenthusiastic, even grudging, acquiescence to the paper's acceptance for publication, some explanation must first be offered as to why, over the years that followed, the paper has had such an impact and, indeed, why it was chosen for inclusion in this issue. Did the referees ‘get it wrong’? While in a sense they *did*, much of the blame for this may be placed on Reynolds' writing: his excessively ‘wordy’ style of communication, his habit of presenting well-established procedures and principles as though they were something new and his use of cumbersome terminology. While Lord Kelvin had introduced the term ‘turbulent flow’ in 1887, Reynolds never adopted this form. Thus he used the terms ‘relative-mean-motion’ and ‘mean-mean-motion’ to denote turbulent and mean velocities respectively. Moreover, the best known output from the paper, the time-averaged equations for a turbulent flow—what are now called the *Reynolds equation*s—didn't in itself lead anywhere; or, at least, was not the subject of any further development by the author. To Reynolds these equations were just a stepping stone to his main goal, the kinetic energy budget for a fluid in turbulent motion and, in particular, how the relative magnitudes of the source and sink terms in that equation changed with Reynolds number. However, it seems probable that if the referees reached page 40 (of the published paper) where the target result appeared, their sensibilities had been dulled to a point where its significance was not appreciated.

Let us now look a little deeper into what the paper contained. In doing so, the writer will adopt Cartesian tensor notation and the compactness afforded by the Einsteinian summation convention [8] which was not available to Reynolds. The first 18 pages of his article are largely taken up with a lengthy pre-statement of the paper's conclusions, with setting out the starting equations, and a discussion on averaging. Reynolds' initial approach to the last of these was via a mass-weighted averaging, a strategy that preceded by 70 years the paper which is usually cited for this ‘novelty’ [9]. However, since his concern was the flow of water, after setting out the mass-weighting principle, he thereafter reverted to what has become the customary volume-based approach (from which density, as a constant, drops out).

On replacing the instantaneous velocity *u*_*i*_ by its mean value, 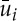 plus a turbulent fluctuating velocity, *u*′_*i*_, he first noted that the continuity equation: ∂*u*_*i*_/∂*x*_*i*_=0 implied that both the mean and the turbulent velocity field were solenoidal, i.e. 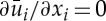 and ∂*u*′_*i*_/∂*x*_*i*_=0. He then proceeded to the momentum equations and likewise obtained (adopting the layout presented by Reynolds):

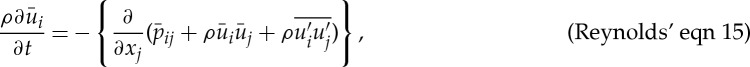

where *ρ* is the fluid density and 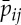 is the mean value of stress applied to the fluid, comprising static pressure and viscous contributions. Reynolds' eqn (15) is known today as the *Reynolds equation* (or ‘equations’ if, as Reynolds, one is using regular Cartesian coordinates) and the quantities 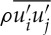 are termed the *Reynolds stresses*, whether in the form shown or with the density omitted (the kinematic form). Of course, before Reynolds' eqn (15) is directly usable, one needs a means for determining the Reynolds stresses in terms of known or calculable quantities. As noted above, Reynolds himself only obliquely touched on this topic. However, over the century following his paper's publication the development of schemes for approximating the Reynolds stresses became a major activity in engineering fluid mechanics. This theme will be returned to below but first the more direct concerns of the paper will be examined.

As noted above, Reynolds' primary interest was to examine the kinetic energy equation for the fluid, including the transfer of energy between the mean and the turbulent motion. To this end he first effectively multiplied Reynolds' eqn (15) by 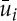. On rearranging the terms, a mean kinetic energy equation was produced:

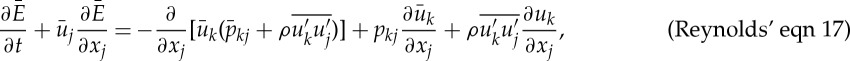

where 
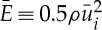
.

He next obtained the momentum equation for the turbulent motion by subtracting the mean momentum equation 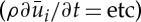 from the instantaneous momentum equation. Then, on multiplying *that* equation by the instantaneous velocity and averaging the result, an equation for the *turbulent* kinetic energy emerged:

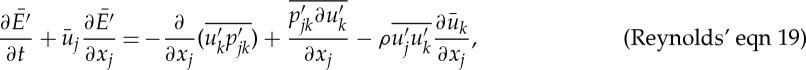

where 
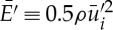
.

At this point a warning is inserted for readers who may wish to follow Reynolds' original analysis in Cartesian coordinates. The equations, besides being considerably longer, also contain many typographical errors, principally associated with the density *ρ* being omitted from many of the terms. The emended version, [10], also reproduced in [11], is thus recommended.

As Reynolds pointed out, the last terms in Reynolds' eqn (17) and Reynolds' eqn (19) were identical save for the difference in sign. The process thus represented a transfer of kinetic energy from the mean motion to turbulence. Before leaving Reynolds' eqn (19), it needs to be underlined what a major achievement its derivation and physical interpretation were. Indeed, it is remarked that the corresponding but simpler equation for the mean-square fluctuating temperature that may be loosely interpreted as the turbulent thermal energy equation (which is closely analogous in its form to that for 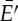, only simpler because of the absence of terms containing pressure) was only published in the 1950s [12].

While many might feel that Reynolds' eqn (19) is essentially the high-point of the paper, for Reynolds it was only a staging point in his journey. It contained diffusive-like terms that could not be handled on a local basis. Thus, for the particular case of fully developed flow through a confined duct, he chose to integrate the equation over the cross section normal to the flow direction. Moreover, at that stage he expressed the stress tensor, *p*_*ij*_ in terms of static pressure and viscous-stress contributions. He thus arrived at the following ‘discriminating equation’ (Reynolds' terminology) that expressed an overall balance between the cross-sectionally averaged generation of turbulence through the interaction of the Reynolds stresses with mean velocity gradients and the destruction of turbulence (again averaged over the cross section) by way of viscous dissipation of the turbulent motion to heat:

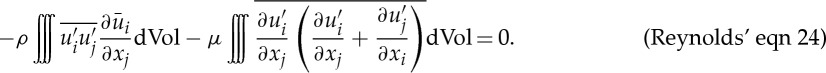



Thereafter, he limited attention to the case of fully developed flow between parallel planes (a configuration that lends itself more readily to analysis than a circular pipe) and assumed certain perturbation forms for the fluctuating velocity field. Finally, after an analysis of several pages, Reynolds arrived at the result that, to maintain a turbulent flow through the passage the Reynolds number should be not less than 517, based on the distance between the planes and the mean velocity averaged over the cross section. While recognizing, of course, that this was little more than one quarter of the value he had determined from his experiments (reported in his 1883 paper), he pointed out that the flow configurations were different and that the actual critical Reynolds number at which turbulent flow would result would be above that value.

## 4. Developments from the 1895 paper

As with many emphatically original contributions, the ideas contained in Reynolds' paper did not immediately take root. The longer term impact of his work is incontrovertible, however.

On the specific topic to which Reynolds' attention had been directed, the prediction of the Reynolds number at which laminar flow would become unstable, over the early decades of the twentieth century analytical strategies considered *infinitesimally* small perturbations about the laminar flow velocity distribution leading to a *linear* stability analysis from which terms involving the nonlinear products of velocity fluctuations (analogous to the Reynolds stresses) were necessarily discarded. This work is summarized in Lin's widely cited textbook [13]. For the case of flow between parallel planes such analyses led to levels of the critical Reynolds number substantially greater than Reynolds' (pipe-flow) experiments [2] had indicated; Stuart [14], for example, cites a critical Reynolds number of 5780. However, from the 1950s onwards, it was acknowledged that the Reynolds stresses needed to be retained—or, equivalently, ‘in a nonlinear theory, the interdependence of the mean and disturbance parts of the flow must be taken into account…’ and that ‘in many cases an equilibrium state may be possible in which the rate of transfer of energy from the (distorted) mean flow to the disturbance balances precisely the energy of dissipation of the energy of disturbance’ [15]. Thus, more than a half-century after the publication of Reynolds' 1895 paper, the advocated strategy returned broadly to what Reynolds had instinctively followed from the outset—albeit with more refined analytical techniques. The first results from this nonlinear analysis appear to be those of Meksyn & Stuart [16], who reported a minimum critical Reynolds number of 2900, again for the case of flow between parallel planes.

Leaping forward to the present, to an energy-conscious world where the prediction (indeed, the prevention where possible) of transition from laminar to turbulent flow is commanding greater and greater attention, one finds the ideas that stimulated Reynolds still the subject of exciting further developments. In March 2014, for example, the conference *Nonlinear Stability Theory: from weakly nonlinear theory to the verge of turbulence* held at the UK's Royal Academy of Engineering, even without formal proceedings, attracted workers from nine countries across four continents to come and share their discoveries.

Turning to the other major issue, that of providing a model for calculating the Reynolds stresses appearing as unknowns in Reynolds' eqn (15), Taylor [17] was among the earliest in proposing strategies for their approximation. Like Prandtl [18], with his mixing-length hypothesis a decade later, attention was limited to simple shear flows where the mean flow travelled broadly in a straight path (*x*_1_, say) and where the velocity varied only in a single direction perpendicular to the flow direction (*x*_2_). In these circumstances only a single component of the Reynolds stress tensor was active 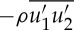 and the chosen strategy was to approximate it in terms of a turbulent eddy viscosity, *μ*_*t*_ : 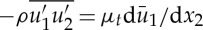. The eddy viscosity was supposed to be linked to a length scale representative of the turbulent eddies and a characteristic turbulent velocity. Thus, the mixing-length hypothesis took 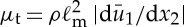 where the mixing length ℓ_*m*_ was prescribed algebraically, linked to physical features of the flow (for example the width of a jet or the distance of a point from a rigid surface). While the mixing-length hypothesis, embedded within numerical solvers of the Reynolds equations, is still used to compute relatively simple boundary-layer flows [19], today the great bulk of computational fluid dynamics (CFD) adopts more elaborate and general practices to approximate the Reynolds stresses. Kolmogorov [20], in a paper that achieved negligible visibility on publication in the war-ravaged Soviet Union, proposed that the characteristic length and velocity scales should themselves be determined by way of transport equations. The turbulent velocity scale was to be determined by solving a transport equation for the turbulent kinetic energy, 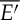 (though nowadays, in CFD circles, the variable universally employed is the kinematic turbulence energy, 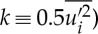. Thus, Reynolds' pioneering research in deriving and elucidating the physical processes present in the turbulent kinetic energy equation significantly contributed to the development of a practical route for characterizing the turbulent mixing processes.

Kolmogorov's paper also proposed that, instead of an *algebraic* prescription of the turbulent length scale, a second scalar property of turbulence should be solved by way of a transport equation from which the effective eddy scale could be obtained. At the time, before the arrival of the digital computer, this idea could not be properly tested. However, the late 1960s and early 1970s saw a burgeoning of such ‘two-equation turbulence models’, e.g. [21–23], that (as the numerical capabilities of computational solvers progressively expanded) were applied to mimic increasingly complex practical turbulent flows.

Success in the computational modelling of such flows was by no means always achieved, however. Besides the considerable empiricism embedded in any of the scale-determining transport equations, the assumption that the behaviour of turbulent flows could be modelled via an assumed eddy viscosity was itself a highly limiting concept that became less and less tenable as attention shifted to flows with increasingly complex strain fields. Indeed, in many cases, there would be the added complication of force-field effects such as buoyancy, too. In fact, from the 1930s, physicists had been seeking ways to approximate the turbulent stresses by routes that did not assume a linear connection between strain rate and Reynolds stress. Proposals were advanced for determining all relevant second- and third-moment turbulent velocity products directly from transport equations, e.g. [24]. There was, however, no prospect (or risk) of these proposals being tested: their advocates were purely armchair explorers! J. C. Rotta is the person who did most to cut these fanciful ideas down to size. He proposed [25] that, in addition to a scale-determining equation, one should simply solve transport equations for the Reynolds stresses. Indeed at least one of his proposed approximations for processes that could not be handled exactly is still widely used today. Current achievements with such models are summarized *inter alia* in a recent textbook [26].

Rotta's level of approximation, known as ‘second-moment closure’, is generally seen today as the most elaborate approach worth following in determining the Reynolds stresses. Several somewhat simpler routes have been proposed, however. Pope [27] and, more comprehensively, Gatski & Speziale [28] discarded or otherwise approximated terms containing derivatives of the Reynolds stresses, thus creating a system of interlinked *algebraic* equations for 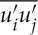 from the erstwhile stress-transport equations. These were then manipulated to produce what amounted to a *nonlinear eddy-viscosity model*. This type of approximation has been increasingly used since the mid-1990s whether formulated by simplifying the stress-transport equations, as above, or by proceeding directly to add quadratic and, sometimes, cubic terms to the constitutive equation linking the Reynolds stress to the mean-flow deformation field, e.g. [29].

As the above summary has endeavoured to show, while Reynolds' original strategy in simplifying the equations of fluid motion for turbulent flow was initially only slowly taken up, it has been at the core of developments in computing the behaviour of practically important turbulent flows for the last century. Indeed, its impact on all our lives is incalculable. The contribution of CFD to the aerodynamics of automobiles, trains and aircraft and, equally, to the power units that drive them, has been and continues to be enormous. On a quite different front, today increasing attention is being given to computing the fluid-flow processes where turbulent flow prevails within the human body to bring us healthier, longer lives.

But what of the future? Will the use of the Reynolds equations survive the twenty-first century or does it face extinction? While this is not the place to attempt a SWOT analysis, it is hardly controversial to observe that two countervailing tendencies will be strongly present, both driven by the continuing growth in and reduction in cost of computing power. On the one hand, the increase in computational resource will mean that for a growing number of applications where improvements in the accuracy of the predictions have substantial benefits to consequent designs or decision making, Reynolds-averaging strategies will progressively be replaced by *large-eddy simulation* of the flows in question. In such schemes, a large proportion of the momentum and heat transport by turbulent motion is directly resolved by the filtered, unsteady equations of motion using a finer (and thus more costly) grid than would be adopted for a Reynolds-averaged computation. Modelling is still required, but only to account for the effects of fine scale turbulence too small to be resolved by the grid adopted. Such approaches first appeared more than 40 years ago and today their mature versions are beginning to be widely applied in the research laboratories from where they will certainly migrate to industrial development centres.

On the other hand, there are many important areas of turbulent flow where Reynolds-averaging approaches are today simply too detailed to be practical or affordable. As computational power becomes greater and cheaper, however, these types of application will progressively begin to look attractive for tackling by way of Reynolds averaging, thereby displacing the integral modelling schemes or other simple approaches that are currently employed. A large proportion of such applications will concern flows in the environment, whether in the atmosphere, rivers or oceans. As examples of potential types of problem, one may cite the discharge of gaseous exhausts from a chemical processing plant or the choice of the optimum placement of wind turbines on a particular site to achieve maximum power output while avoiding damaging mutual interference of the turbines. As a somewhat later application, one may hope to provide detailed predictions of the birth and initial growth of cyclones with a view to seeking ways to limit their lethal impacts. Thus, while the types of flow tackled will continually change over the twenty-first century, it seems highly probable that, throughout it, the world will continue to derive benefit from Osborne Reynolds' legacy.

## Author profile

**Brian Launder's** research has been closely associated with the measurement and modelling of turbulent flow throughout his career. He had obtained his first degree in mechanical engineering from Imperial College, London, an institution he rejoined for 12 years after completing master's and doctoral degrees at MIT for research on boundary-layer laminarization. After a 4-year spell as professor at the University of California, Davis, he moved to Manchester, heading the Thermo-Fluids group at UMIST. In 1994, he was admitted a Fellow of both the Royal Society and the Royal Academy of Engineering for his research on turbulent flows. Following the merger of UMIST with the Victoria University of Manchester Brian's interests extended to biographical accounts of distinguished professors from the early years of the university, including, especially, Osborne Reynolds about whom he has written several articles.
